# The SPP1-CD44 Signaling Axis Orchestrates Macrophage Metabolism to Promote Early Inflammation in Acute Kidney Injury

**DOI:** 10.7150/ijbs.130922

**Published:** 2026-05-01

**Authors:** Na Gong, Wenjuan Wang, Yifei Fu, Xinru Guo, Xumin Zheng, Yanjun Liang, Yuhao Chen, Yan Chen, Shengchun Zheng, Xiangmei Chen, Guangyan Cai

**Affiliations:** 1 Medical School of Chinese PLA, Beijing, 100853, China.; 2 Department of Nephrology, First Medical Center of Chinese PLA General Hospital, State Key Laboratory of Kidney Diseases, National Clinical Research Center for Kidney Diseases, Beijing Key Laboratory of Medical Devices and Integrated Traditional Chinese and Western Drug Development for Severe Kidney Diseases, Beijing Key Laboratory of Digital Intelligent TCM for the Prevention and Treatment of Pan-vascular Diseases, Key Disciplines of National Administration of Traditional Chinese Medicine(zyyzdxk-2023310), Beijing 100853, China.; 3 Department of Nephrology, Characteristic Medical Center of Chinese People's Armed Police Force, Tianjin 300162, China.

**Keywords:** acute kidney injury, intercellular communication, macrophage, metabolic reprogramming

## Abstract

The extent of the inflammatory response in the early stages of acute kidney injury (AKI) significantly influences renal damage, repair, and ultimately prognosis. Macrophages are key drivers of early inflammation in AKI, and their metabolic reprogramming is closely associated with their pro-inflammatory polarization. However, the mechanisms underlying this process remain incompletely understood. In this study, we combined single-cell RNA sequencing, metabolomics, and gene-editing approaches to, investigate how injured renal tubular epithelial cells regulate macrophage metabolism and phenotype in ischemia-reperfusion injury (IRI)-induced AKI. We found that injured proximal tubular cells secrete high levels of secreted phosphoprotein 1 (SPP1), which binds to CD44, a receptor abundantly expressed on infiltrating macrophages, thereby activating the downstream PI3K/AKT signaling pathway. This activation induces nuclear translocation of PKM2, a key metabolic enzyme, which drives glycolytic metabolic reprogramming in macrophages and promotes their polarization toward a pro-inflammatory phenotype. *In vitro* and *in vivo* functional experiments further confirmed that blocking the SPP1-CD44 axis, using siRNA, neutralizing antibodies, or conditional knockout strategies, effectively alleviates renal IRI in mice, reduces macrophage infiltration, and diminishes the inflammatory response. Overall, this study delineates a novel mechanism in which injured tubular cell-derived SPP1 communicates with macrophage CD44 to regulate immunometabolism and inflammatory polarization via the PI3K/AKT-PKM2 signaling module at the single-cell and metabolic levels. These findings provide both a potential therapeutic target and a mechanistic framework for the prevention and treatment of AKI.

## 1. Introduction

Acute kidney injury (AKI) is a common clinical condition that frequently progresses to chronic kidney disease. Currently, there are no specific therapies for AKI, creating a substantial burden on healthcare systems worldwide 1. Ischemia-reperfusion injury (IRI) is one of the major etiologies of AKI. Consistent with previous studies, our preliminary research has also found that inflammatory responses play a central role in driving the imbalance between renal tubular injury and repair 2. Macrophages are highly plastic immune cells that exhibit pleiotropic and coordinated responses to tissue microenvironmental signals at different stages of AKI. Our previous single-cell RNA sequencing (scRNA-seq) analysis revealed that Treml4^+^ monocytes differentiate into pro-inflammatory Fn1^+^ macrophages after infiltrating IRI-injured renal tissues 3. Our previous review summarizes that, during the early stages of AKI, macrophages predominantly exhibit pro-inflammatory characteristics and undergo metabolic reprogramming involving glycolysis and the pentose phosphate pathway. As AKI progresses, these macrophages gradually shift toward an anti-inflammatory phenotype, relying primarily on oxidative phosphorylation 4. This metabolic flexibility allows macrophages to adapt to tissue injury and the inflammatory microenvironment, thereby modulating the early inflammatory response in AKI.

In the tissue microenvironment of IRI-induced AKI, cell-cell interactions mediate the early immune-inflammatory response and injury repair in AKI. Consistent with previous studies, we observed prominent renal tubular injury at the cortico-medullary junction, accompanied by the secretion of extracellular vesicles enriched in integrin β1. These vesicles recruit numerous immune cells, particularly macrophages, thereby promoting renal inflammation and pathological repair 3. Subsequently, based on scRNA-seq results, we further characterized the heterogeneity of injured proximal tubular (PT) cells. Among these populations, injured PT cells exhibited significantly increased secreted phosphoprotein 1 (SPP1) expression. Further cell-cell interaction analysis revealed that SPP1-CD44 is the key ligand-receptor pair mediating crosstalk between injured PT cells and monocyte/macrophages. These findings indicate that injured PT cells may regulate macrophage behavior through the SPP1-CD44 axis. However, the molecular mechanisms underlying this regulation remain unclear.

SPP1 is a secreted phosphoprotein that is highly expressed in various inflammatory and fibrotic diseases 5, 6. Previous clinical studies have demonstrated that SPP1 levels are significantly elevated in the plasma, urine, and renal cortex of patients with multiple kidney diseases, including diabetic nephropathy and lupus nephritis, and that these correlate with disease severity, suggesting a potential role for SPP1 in renal pathological progression 7, 8.

CD44 is a membrane-bound glycoprotein expressed on various inflammatory cells and participates in numerous pathophysiological processes, including cell adhesion, migration, cytoskeletal reorganization, metabolism, inflammation, proliferation, and angiogenesis. Previous studies have shown that the CD44 expression can negatively regulate PKM2 kinase activity, promotes glycolysis, and contribute to tumor initiation and progression 9. Therefore, this study focuses on determining whether the SPP1-CD44 axis mediates the metabolic reprogramming of macrophages regulated by injured PT cells.

Accordingly, based on analyses of intercellular communication derived from previous scRNA-seq data, this study systematically elucidates the upstream signaling mechanisms by which injured PT cells in IRI-induced AKI drive macrophage metabolic reprogramming and pro-inflammatory phenotypic transformation. First, this study investigates whether SPP1-CD44-mediated intercellular interactions exist between injured renal tubules and macrophages by integrating scRNA-seq with functional experiments. Next, using *in vitro* gene knockdown approaches, this study explores the specific mechanisms by which the SPP1-CD44 axis regulates macrophage metabolic reprogramming and phenotypic transformation. Finally, through neutralizing antibody intervention and conditional knockout of renal tubular epithelial cells *in vivo*, we examine whether blocking the SPP1-CD44 axis effectively alleviates kidney injury and macrophage-mediated excessive inflammation. Collectively, this study provides critical insights into intercellular communication mechanisms within the AKI tissue microenvironment and lays an essential foundation for the future development of novel therapeutic strategies.

## 2. Materials and Methods

### 2.1 Animals

Male C57BL/6J mice (6-8 weeks old) were housed under specific pathogen-free conditions. All experimental procedures were reviewed and approved by the Animal Ethics Committee of the General Hospital of the People's Liberation Army (Ethics approval number: 2024-X20-113). The renal IRI model was established by clamping the bilateral renal pedicles for 30 min, followed by restoration of blood flow. Mice in the sham-operated group underwent the same surgical procedures without arterial clamping. At 24 h post-reperfusion, mouse serum samples were collected, and serum creatinine (Scr) and blood urea nitrogen (BUN) levels were measured using commercial biochemical assay kits (Nanjing Jiancheng) to evaluate renal function. Meanwhile, kidney tissues were fixed, paraffin-embedded, and sectioned for hematoxylin and eosin (H&E) and periodic acid-Schiff (PAS) staining. For neutralizing antibody intervention, anti-SPP1 antibody, anti-CD44 antibody, or the corresponding control IgG antibody (10 mg/kg, BioXCell) was administered via intraperitoneal injection immediately at the onset of reperfusion.

This study generated mice with conditional knockout of *SPP1* specifically in renal tubular epithelial cells. The information of the tool mouse strains used is as follows: *SPP1* flox^+/+^ homozygous mice carrying loxP sites flanking exons 2-8 of the *SPP1* gene, and mice expressing proximal tubule-specific Cre recombinase (*Cdh16-CreERT2*), both purchased from GemPharmatech Co., Ltd. (Jiangsu, China). *SPP1* flox^+/+^ mice were crossed with Cre-positive mice to obtain the experimental genotypes: *SPP1* flox^+/+^; Cre^+/-^ (conditional knockout, CKO) and their littermate controls *SPP1* flox^+/+^; Cre^-/-^ (Control). To induce specific knockout of *SPP1* in renal tubular epithelial cells, CKO and control mice received intraperitoneal injections of tamoxifen (100 mg/kg, administered every other day, for a total of three doses). Following tamoxifen administration, all mice were allowed to recover for at least 7 days before undergoing ischemia-reperfusion modeling.

### 2.2 Cell culture

The mouse macrophage cell line Raw264.7 (Genepharma) was cultured in high-glucose DMEM supplemented with 10% fetal bovine serum and 1% penicillin/streptomycin (Gibco) at 37 °C under 5% CO₂. All intervention experiments were performed using cells in the logarithmic growth phase. For gene knockdown, small interfering RNA (siRNA) constructs from Genepharma were transfected at a concentration of 50 nM using transfection reagent (Dona Pharmaceutical) 24 h prior to stimulation with recombinant SPP1 protein (R&D Systems). For signaling pathway intervention experiments, cells were pretreated for 12 h with either a PI3K/AKT pathway inhibitor (Selleck) or the PKM2 tetramerization activator TEPP-46 (Selleck) prior to SPP1 stimulation, at final concentrations of 1 μg/ml and 10 μg/ml, respectively.

Primary renal tubular epithelial cells were isolated from the renal cortical tissues of C57BL/6 mice. The cortical tissues were minced and digested with collagenase type I (Gibco) at 37 °C for 35 min to obtain a single-cell suspension. The digested suspension was sequentially filtered through 100 μm and 40 μm cell strainers to remove undigested tissue clumps. Subsequently, red blood cell contamination was eliminated via treatment with red blood cell lysis buffer, and cell pellets were collected by centrifugation. The cells were resuspended in DMEM/F12 complete medium supplemented with 10% fetal bovine serum, 20 ng/ml epidermal growth factor (EGF, PeproTech) and 1× insulin-transferrin-selenium (ITS, Pricella), followed by incubation at 37 °C in a humidified atmosphere containing 5% CO₂. The first medium change was performed 48 h after seeding. Prior to hypoxia treatment, the cells were synchronized for 12 h in serum-containing, glucose-free medium. During hypoxia induction, the culture medium was replaced with serum- and glucose-free medium. Following hypoxia, the cells were reoxygenated and cultured again in normal complete medium.

### 2.3 Quantitative real-time PCR analysis

Total cellular RNA was extracted using the Trizol method, followed by cDNA synthesis with the HiScript IV RT SuperMix for qPCR (+gDNA wiper) reverse transcription kit (Vazyme Biotech Co., Ltd.). Subsequently, quantitative real-time PCR was performed using the Taq Pro Universal SYBR qPCR Master Mix (Vazyme Biotech Co., Ltd.) in accordance with the manufacturer's standard protocol. Gene expression levels were calculated and analyzed via the 2^(-ΔΔCt) method, and the primer sequences used are detailed in Supplementary Table 1.

### 2.4 Western blotting

Total proteins were extracted from the collected cellular or tissue samples using RIPA lysis buffer, and the protein concentration was quantified via the BCA method. Equal amounts of protein were separated using SDS-PAGE and then transferred onto nitrocellulose membranes. After blocking with 5% non-fat milk for 2 h, the membranes were sequentially incubated with specific primary antibodies (at 4 °C overnight) and the corresponding secondary antibodies (at room temperature for 2 h). Finally, protein bands were visualized and imaged using an enhanced chemiluminescence system. The information of primary antibodies used is as follows: SPP1, Kim-1, PKM2, iNOS and IL-1β (Proteintech); AKT and p-AKT (Cell Signaling Technology); CD44 (Abcam); TNF-α (WanleiBio); and β-actin (Servicebio).

### 2.5. Immunohistochemistry (IHC) and immunofluorescence (IF)

IHC: For paraffin-embedded sections, deparaffinization and rehydration were performed using xylene and a graded ethanol series, respectively. Following antigen retrieval and quenching of endogenous peroxidase activity with 3% H₂O₂, non-specific binding sites were blocked with goat serum. Sections were incubated with primary antibodies at 4 °C overnight, followed by incubation with appropriate secondary antibodies at room temperature the next day. Signal detection was performed using DAB, and nuclei were counterstained with hematoxylin.

IF on Tissue: After thawing, frozen tissue sections underwent permeabilization with 0.2% Triton X-100 and blocking with goat serum. Sections were incubated with primary antibodies at 4 °C overnight, followed by fluorophore-conjugated secondary antibodies at room temperature. Nuclei were stained with DAPI.

IF on Cells: RAW264.7 cells were seeded on confocal dishes. After experimental treatments, cells were fixed with 4% paraformaldehyde for 15 min at room temperature, washed with PBS, and then blocked and permeabilized for 1 h using a solution containing 0.2% Triton X-100 and goat serum. Subsequently, cells were incubated with primary antibodies overnight at 4 °C, followed by incubation with Cy3- (1:200, Beyotime) or FITC-labeled (1:50, Beyotime) secondary antibodies in the dark for 2 h. Nuclei were stained with DAPI before mounting. Images were captured using a FV3000 confocal microscope.

The primary antibodies and their dilutions used in this study were as follows: CD44 (1:4000, Abcam), CD68 (1:100, Abcam), SPP1 (1:400, Proteintech), PKM2 (1:200, Proteintech), and LTL (1:200, Vector Laboratories).

### 2.6 Flow cytometry

RAW264.7 cells were collected and resuspended in PBS containing 2% FBS. For surface marker staining, cells were incubated in the dark with PE/Cy7-conjugated anti-F4/80 antibody (BioLegend) and APC-conjugated anti-CD86 antibody (BioLegend), both at a concentration of ≤0.25 µg per million cells (in a 100 µL reaction volume). For intracellular detection of tumor necrosis factor-α (TNF-α), cells were treated with a fixation/permeabilization buffer (BioLegend) prior to staining with the appropriate fluorescently-labeled antibody. ROS levels were measured by incubating cells with the fluorescent probe DCFH-DA (1:1000, Servicebio) in the dark. For cell cycle analysis, cells were fixed in pre-chilled 70% ethanol at 4 °C for 2 h and subsequently stained with propidium iodide staining solution (Beyotime). After all incubation steps, samples were washed three times with PBS containing 2% FBS, resuspended in an appropriate volume of buffer, and analyzed using a flow cytometer.

### 2.7 Seahorse

Real-time cellular metabolic phenotyping was performed using the Seahorse XFe24 Extracellular Flux Analyzer (Agilent). The cell culture plate was coated with poly-L-lysine (Beyotime) 3 h prior to the assay. Subsequently, Raw264.7 cells were seeded at a density of 8 × 10⁴ cells per well and cultured overnight. After cell attachment, the corresponding gene knockdown or recombinant SPP1 protein stimulation was applied. Immediately before the assay, the medium was replaced with XF DMEM assay medium (Alicelligent) supplemented with 10 mmol/L glucose, 2 mmol/L glutamine, and 1 mmol/L sodium pyruvate. Following the manufacturer's protocol, 1.5 μmol/L Oligomycin, 0.75 μmol/L FCCP, 0.5 μmol/L Rotenone/Antimycin A, and 50 mmol/L 2-Deoxy-D-glucose were sequentially injected. Following the measurement, the obtained data were normalized to either final cell counts or total protein content.

### 2.8 Wound-healing assay

RAW264.7 cells were seeded in 6-well plates. When the cells reached 80%-90% confluence, a straight scratch was created using a sterile 200 µL pipette tip. After washing with PBS, the medium was replaced with serum-free medium. Images of the same field were captured under a microscope at 0, 12, 24, and 48 hours post-scratching. The scratch area was quantified using Image J software.

### 2.9 Transcriptomic and metabolomic sequencing

For transcriptomic analysis, total RNA was extracted from RAW264.7 cells after SPP1 stimulation using TRIzol reagent (Thermo Fisher Scientific). Following reverse transcription, RNA-seq was performed on an Illumina high-throughput sequencing platform at MetWare Biotechnology (Wuhan, China). Differential expression analysis between sample groups was conducted using the DESeq2 package. Genes with an absolute log2 fold change ≥ 1 and a false discovery rate < 0.05 were considered differentially expressed. Enriched biological processes were analyzed via Kyoto Encyclopedia of Genes and Genomes (KEGG) pathway enrichment and Reactome analyses.

For metabolic profiling, metabolites were detected using liquid chromatography-tandem mass spectrometry. Metabolites with a Variable Importance in Projection score > 1 and a P-value < 0.05 were identified as significantly altered.

### 2.10 Analyses of scRNA-seq data

The single-cell transcriptome data analyzed in this study were obtained from our previously established and published dataset of renal ischemia-reperfusion injury^3^**.** Single-cell transcriptome data from previous studies were analyzed using the Seurat R package (v4.0.3) in this study. CellChat was applied to perform intercellular communication analysis on annotated cell subsets, which enabled the identification of the global landscape of ligand-receptor-mediated intercellular communication among subsets based on single-cell data, followed by comparison of intercellular communication profiles between the two groups of samples. Subsequently, the global information flow within individual signaling pathways was compared to identify differential signaling pathways that were closed, decreased, opened, or increased between the Sham and AKI groups. The results were visualized in three modes (outgoing signaling, incoming signaling, and overall signaling), to identify signaling pathways and ligand-receptor pairs with distinct signaling patterns. Finally, differences in ligand-receptor pairs between different cell subsets were compared to screen for differential ligand-receptor pairs between groups. Additionally, scMetabolism was used to calculate metabolic pathway activity scores and visualize the scores across all cells, thus rapidly depicting the metabolic landscape.

### 2.11 Enzyme-linked immunosorbent assay (ELISA)

The concentration of SPP1 was measured using a sandwich ELISA kit (Proteintech). Briefly, standards and cell culture supernatants were added to a 96-well plate pre-coated with a capture antibody. After incubation, a biotin-conjugated detection antibody was added. Following further incubation and washes, horseradish peroxidase-labeled streptavidin was applied. The plate was incubated again, washed, and then incubated with TMB substrate solution for color development. The reaction was stopped with stop solution. Absorbance was immediately measured at 450 nm using a microplate reader, and the target protein concentration in the samples was calculated based on the standard curve.

### 2.12 Electron microscopy

Treated cells were collected and pre-fixed with 2.5% glutaraldehyde, followed by post-fixation with 1% osmium tetroxide. After dehydration through a graded ethanol series, the samples were embedded in epoxy resin. Ultrathin sections were prepared, double-stained with uranyl acetate and lead citrate, and then observed under a transmission electron microscope to capture images of the cellular ultrastructure.

### 2.13 Lactate assay

Lactate levels were measured using a commercial assay kit (Servicebio). Cell culture supernatants or serum were collected and working solutions were prepared according to the manufacturer's instructions. Samples, standards, and blank controls were added to a 96-well plate, followed by the addition of the working solution. After thorough mixing, the reaction proceeded at 37 °C for 5 min. Absorbance was measured at 530 nm using a microplate reader. The lactate concentration in the samples was calculated based on the standard curve generated from the absorbance values of the lactate standards.

### 2.14 Statistical analysis

Statistical analyses were performed using GraphPad Prism software (version 10.1.2). Comparisons between two groups were conducted using the unpaired Student's t-test, while comparisons among multiple groups were performed using one-way analysis of variance. A P-value of less than 0.05 was considered statistically significant.

## 3. Results

### 3.1. SPP1 pathway is involved in intercellular communication during IRI

**Figure 1:**
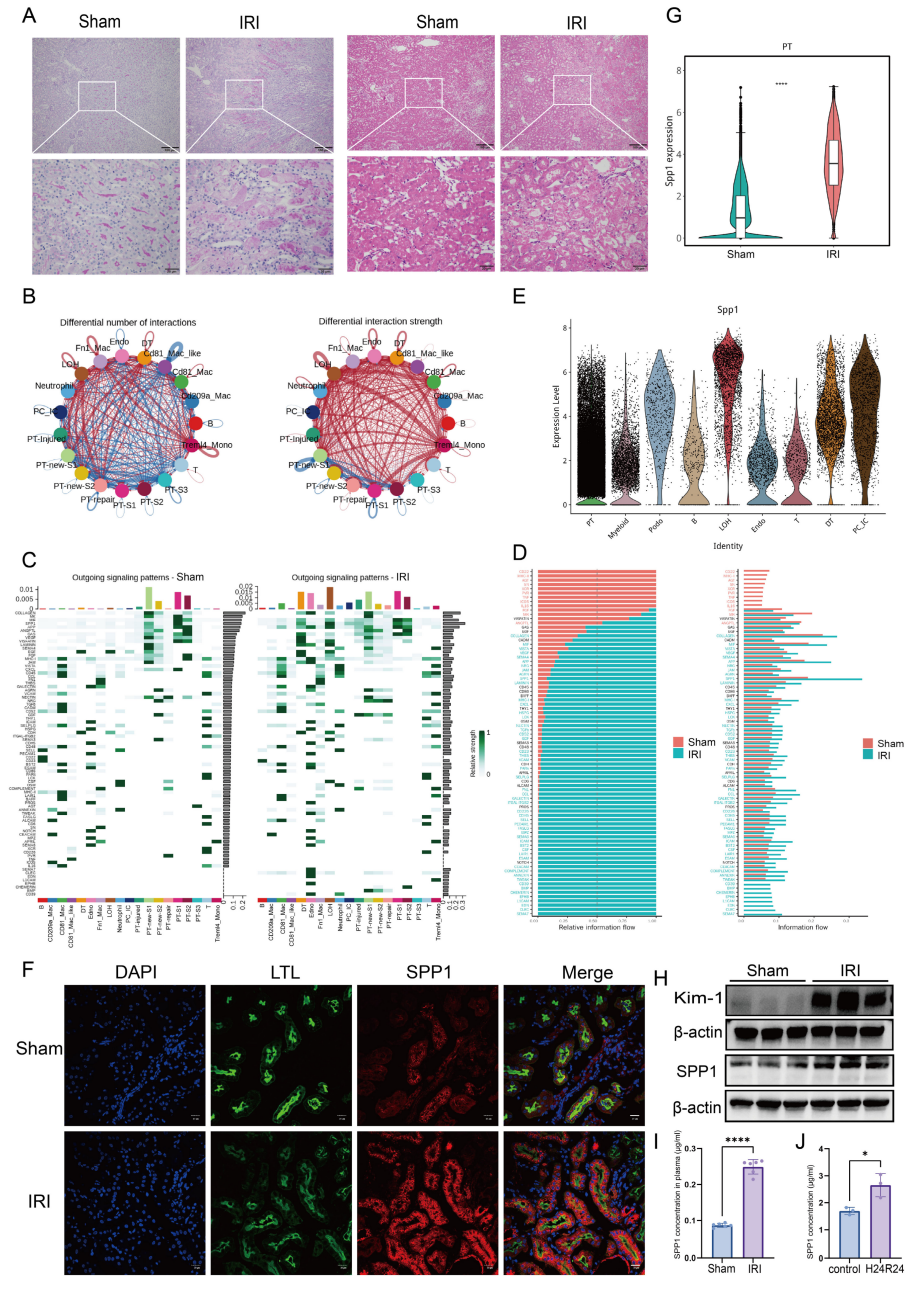
** SPP1 pathway is involved in intercellular communication during IRI.** (**A**) Histopathological assessment of renal tissue following ischemia-reperfusion injury (IRI) using hematoxylin and eosin (H&E) and periodic acid-Schiff (PAS) staining. (**B**) Schematic representation of the number and strength of intercellular ligand-receptor interactions in the kidney post-IRI. (**C**) Analysis of activated outgoing signaling pathways across different cell subpopulations in the Sham and IRI groups. (**D**) Comparison of signaling pathway activity between the Sham and IRI groups. (**E**) Expression of secreted phosphoprotein 1 (SPP1) across different cell subpopulations. (**F**) Immunofluorescence colocalization of SPP1 and LTL in the Sham and IRI groups. (**G**) Comparison of SPP1 expression levels in proximal tubular (PT) cells between the Sham and IRI groups. (**H**) Protein expression levels of Kim-1 and SPP1 in the Sham and IRI groups. (**I**) Serum SPP1 concentrations in the Sham and IRI groups. (**J**) SPP1 secretion in culture supernatants following hypoxia/reoxygenation.

Initial assessment of renal function markers, including Scr and BUN, in mice subjected to renal IRI revealed a significant increase in Scr levels, indicating substantial renal impairment post-IRI (Fig. S1A). Histological evaluation using H&E and PAS staining further demonstrated that the most severe tubular damage occurred at the corticomedullary junction following IRI, accompanied by prominent immune cell infiltration (Fig. 1A). The colocalization of damaged tubular epithelial cells and infiltrating immune cells suggests a potential interplay between these cell types.

To dissect the underlying intercellular communication, ligand-receptor interactions were analyzed using existing single-cell transcriptomic data. We compared the number and interaction strength of ligand-receptor pairs between the Sham and IRI groups. While no significant difference was observed in the total number of interactions, their interaction strength was markedly enhanced in the IRI group (Fig. 1B; Fig. S1B). Specifically, the number of ligand-receptor pairs significantly increased between several cell subsets—including Treml4⁺ monocytes, Fn1⁺ macrophages, distal tubule (DT) cells, loop of Henle (LOH) cells, and multiple PT subpopulations. The strength of ligand-receptor binding was notably intensified not only between LOH and multiple PT subpopulations (including PT-injured), but also between these tubular subsets and myeloid cells, particularly Treml4⁺ monocytes, CD81⁺ macrophages, and Fn1⁺ macrophages (Fig. 1B).

To further analyze the directionality of signaling, patterns from the perspectives of outgoing, incoming, and overall signaling were examined. In the outgoing signaling analysis, compared to that in the Sham group, several resident renal cell types—including LOH, DT, endothelial (Endo), PT-injured, PC-IC, and PT-repair cells—exhibited markedly activated signaling pathways in the IRI group. Among these, PT-injured cells primarily signaled via pathways involving SPP1, APP, GAS, and GDF (Fig. 1C). Incoming signaling analysis indicated that Treml4⁺ monocytes, CD81⁺ macrophages, LOH, Fn1⁺ macrophages, DT, and Endo received increased incoming signals, whereas signals received by PT-new-S1, PT-new-S2, PT-S1, and PT-S2 were reduced compared to those in the Sham group. Notably, pathways associated with collagen, MK, SPP1, THBS, and FN1 were significantly activated in LOH cells (Fig. S1C). Overall signaling analysis further revealed significant activation of pathways involving MIF, SPP1, VISFATIN, BST2, ARIL, Chemerin, and CD39 in Fn1⁺ macrophages, as well as collagen, MIF, SPP1, APP, ANGPTL, and Sema4 pathways in PT-injured cells (Fig. S1D). Subsequently, we compared the overall information flow of each signaling pathway (Fig. 1D). Pathways mediated by CD22, MHC-II, AGT, SN, XCR, PVR, TNF, ICOS, and IL-16 were specifically active in the Sham group, whereas pathways involving SEMA7, CLEC, EDN, L1CAM, EPHB, Chemerin, BMP, and CD39 were inactive in the IRI group. Importantly, signaling mediated by SPP1 accounted for the largest proportion in the IRI group.

Consequently, the changes in SPP1 expression at the genetic, protein, and histopathological levels were evaluated. Tracing the cellular origin of SPP1 revealed that PTs were the primary source of SPP1 expression following IRI (Fig. 1E). Immunofluorescence colocalization confirmed that LTL⁺ PT cells were a major cell type expressing SPP1 (Fig. 1F). Intergroup comparisons revealed that *SPP1* gene expression in PTs, protein levels in tissue, serum concentrations in mice, and expression of the tubular injury marker Kim-1 were all significantly higher in the IRI group than those in the Sham group (Fig. 1G-I; Fig. S1H). Similarly, in primary renal tubular epithelial cells subjected to 24 h of hypoxia/reoxygenation, SPP1 secretion into the culture supernatant was markedly increased compared to that secreted by cells maintained under normal conditions (Fig. 1J).

Collectively, the single-cell transcriptomic analysis reveals an overall enhancement and remodeling of the intercellular communication network following renal IRI and suggests that injured PT cells may interact with other cell types via SPP1.

### 3.2. SPP1-CD44 axis mediates the interaction between injured PT cells and macrophages following IRI

**Figure 2:**
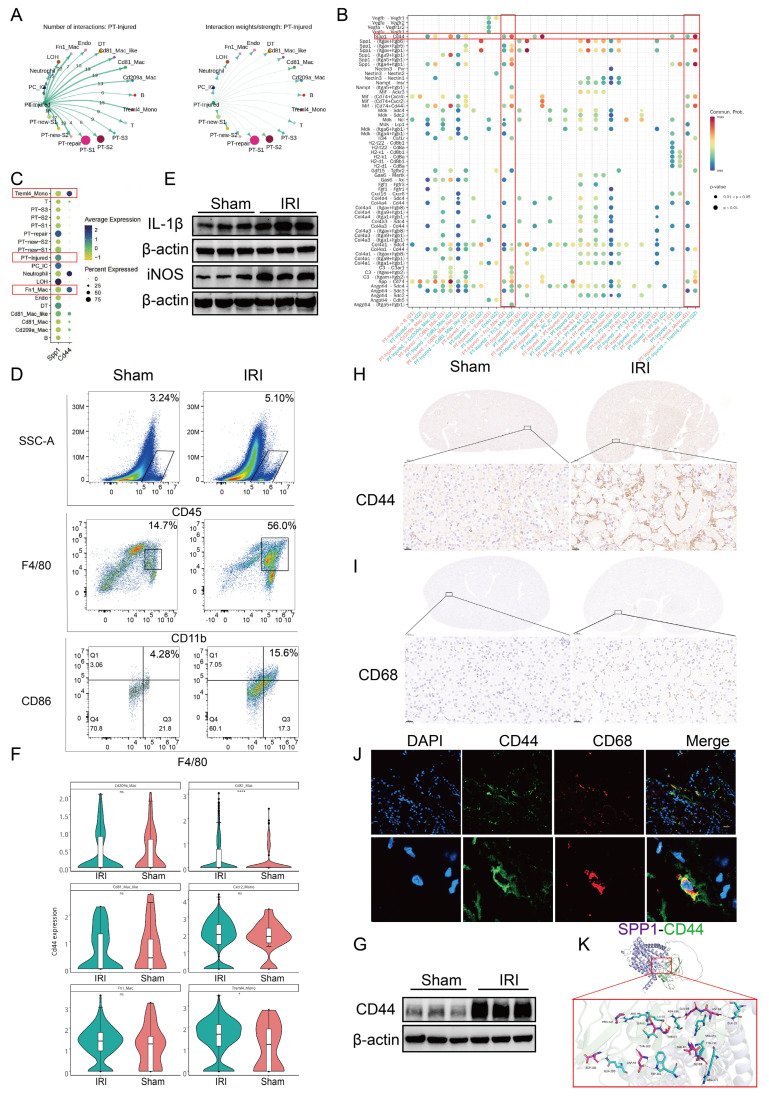
** SPP1-CD44 axis mediates the interaction between injured proximal tubular (PT) cells and macrophages following ischemia-reperfusion injury (IRI).** (**A**) Analysis of the number and strength of interactions between PT-injured subpopulations and other cell types. (**B**) Analysis of ligand-receptor signaling pairs mediating interactions between PT-injured subpopulations and other cell types. (**C**) Cellular source analysis of SPP1 and its receptor CD44. (**D**) Flow cytometric analysis of immune cell infiltration in the kidneys of Sham and IRI mice. (**E**) Protein expression levels of interleukin-1 beta (IL-1β) and iNOS in kidneys from the Sham and IRI groups. (**F**) *CD44* expression across myeloid cell subpopulations based on single-cell RNA sequencing data. (**G**) CD44 protein expression in kidneys from the Sham and IRI groups. (**H**) Immunohistochemical analysis of CD44 expression in kidney tissues from the Sham and IRI groups. (**I**) Immunohistochemical analysis of macrophage infiltration in kidneys from the Sham and IRI groups. (**J**) Immunofluorescence staining of CD44 expression on CD68⁺ macrophages. (**K**) Molecular docking simulation of the interaction between SPP1 and CD44.

To clarify which cell types interact with injured PT cells post-IRI, CellChat was used to analyze the number and strength of their interactions. PT-injured cells formed a greater number of communication links with myeloid cell subsets, including Fn1⁺ macrophages, CD81⁺ macrophages, CD81⁺ Mac-like cells, and Treml4⁺ monocytes. Among these, the number of ligand-receptor pairs between PT-injured cells and Fn1⁺ macrophages was the highest. However, the overall interaction strength (weight) of these ligand-receptor pairs between PT-injured cells and the myeloid cell subsets did not differ significantly (Fig. 2A).

Next, we used bubble plots to illustrate the differential activity of ligand-receptor pairs across distinct cell subpopulations. Pathways involving SPP1-CD44, SPP1-(Itga4 + Itgb1), SPP1-(Itga5 + Itgb1), SPP1-(ItgaV + Itgb1), SPP1-(ItgaV + Itgb5), MIF-(CD74 + CD44), and APP-CD74 were significantly activated (Fig. 2B).

Building on the finding that SPP1 was the top-ranked pathway mediating intercellular communication (Fig. 1), we employed circos and bar plots to visualize the interactions mediated by SPP1 as a ligand and their relative proportions (Figs. S2A, S2B). Among these, signaling via SPP1-CD44 primarily mediated interactions between PT and immune cells, particularly macrophages.

Next, we traced the cellular origins of SPP1 and CD44, which revealed that SPP1 was primarily secreted by injured PT cells (including PT-repair and PT-injured subtypes) and LOH cells, whereas CD44 was predominantly expressed on myeloid cells, including Fn1⁺ macrophages, Treml4⁺ monocytes, and neutrophils. These findings further support the role of the SPP1-CD44 axis in mediating interactions between injured PT cells and pro-inflammatory myeloid cells (Fig. 2C).

The role of the SPP1-CD44 axis was further validated at the histopathological, cellular, and inflammatory levels. Flow cytometry analysis showed that the numbers of total CD45⁺ leukocytes, CD11b⁺ myeloid cells, and CD86^+^ macrophages/monocytes (indicative of activation) were higher in the kidneys of IRI mice compared to those in the Sham mice (Fig. 2D; S2C). Western blot analysis revealed a marked upregulation of the pro-inflammatory cytokines IL-1β and iNOS following IRI (Fig. 2E; S2D), indicating an early increase in pro-inflammatory macrophage infiltration.

Subsequently, using scRNA-seq data, the differential expression of CD44 in inflammatory cells was analyzed. CD44 was broadly upregulated in myeloid cells, with particularly high expression in Treml4⁺ monocytes and CD81⁺ macrophages (Fig. S2E; 2F). Western blot analysis confirmed that CD44 protein expression in renal tissue was significantly elevated in the IRI group compared to that in the Sham group (Fig. 2G, S2F). IHC further showed a pronounced increase in CD44 expression within the renal interstitium. Similarly, CD68⁺ macrophages were primarily localized in the interstitial region (Fig. 2H-I; S2G-I). Immunofluorescence staining showed colocalization of CD68 and CD44, indicating that CD68⁺ macrophages express CD44 (Fig. 2J). Finally, molecular docking simulations indicated that SPP1 and CD44 form extensive complementary contact interfaces on their protein surfaces. Multiple key amino acid residues stabilize this interaction via a hydrogen bond network, with a ZDOCK score of 20.58, suggesting a high-affinity binding between the two molecules (Fig. 2K).

### 3.3. SPP1 promotes macrophage pro-inflammatory polarization, oxidative stress, and migration

**Figure 3:**
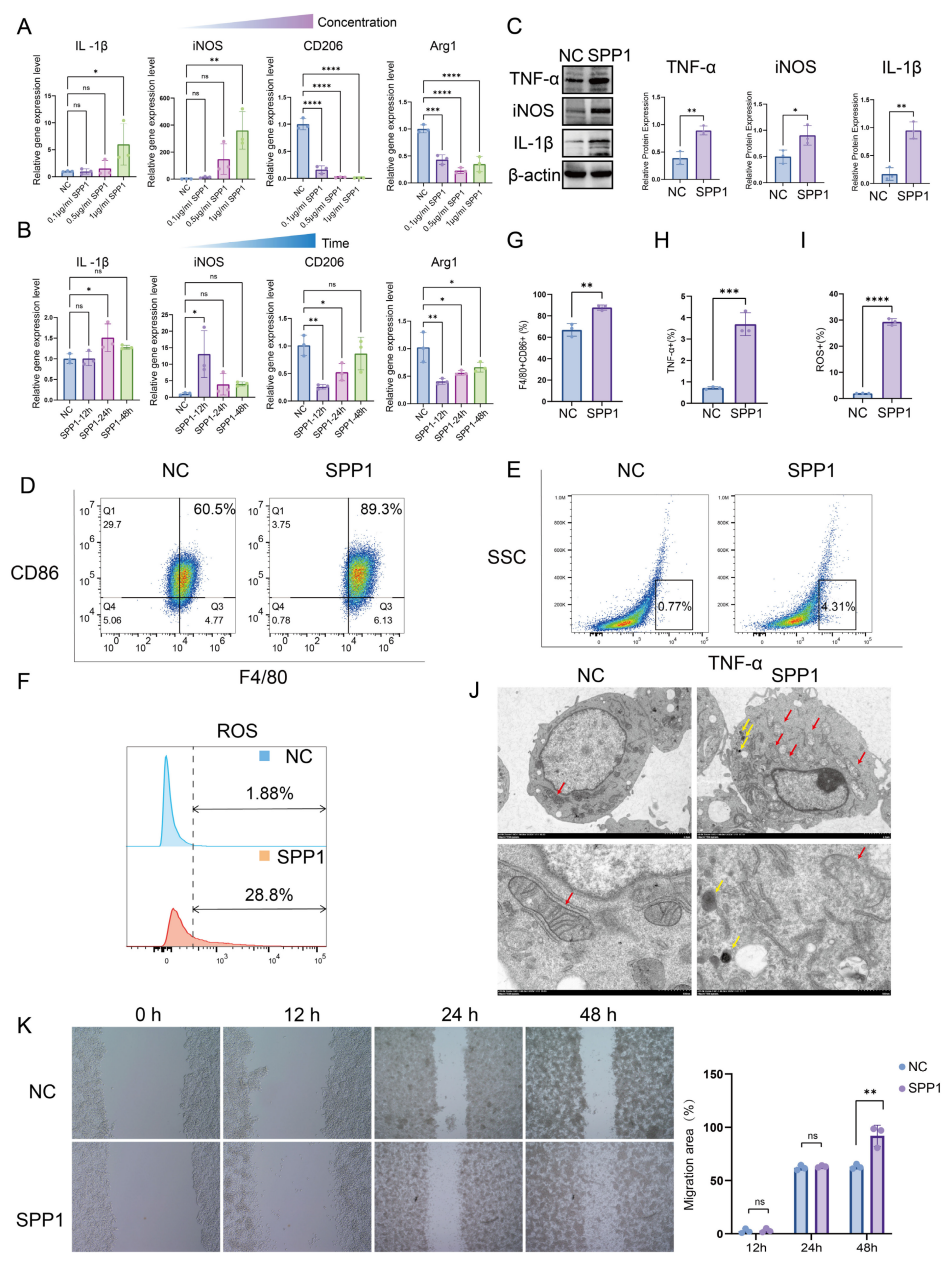
** SPP1 promotes macrophage pro-inflammatory polarization, oxidative stress, and migration.** (**A** and **B**) Dose- and time-dependent effects of SPP1 stimulation on the expression of pro- and anti-inflammatory genes in macrophages, assessed via quantitative polymerase chain reaction (qPCR). (**C**) Effects of SPP1 stimulation on the protein expression of tumor necrosis factor alpha (TNF-α), inducible nitric oxide synthase (iNOS), and interleukin-1 beta (IL-1β) in macrophages via western blotting. (**D**-**I**) Effects of SPP1 stimulation on macrophage phenotypes (CD86 and TNF-α) and reactive oxygen species levels, with quantitative analysis via flow cytometry. (**J**) Effects of SPP1 stimulation on macrophage ultrastructure (mitochondria: red arrows, autolysosome: yellow arrows), evaluated using transmission electron microscopy. (**K**) Effects of SPP1 stimulation on macrophage migratory capacity, assessed through wound-healing assays.

To clarify the regulatory role of SPP1 in macrophage function, macrophages were stimulated with recombinant SPP1 protein at varying concentrations (0, 0.1, 0.5, and 1.0 μg/mL) and for different durations (0, 12, 24, and 48 h). qPCR analysis demonstrated that SPP1 substantially upregulated the expression of pro-inflammatory genes (*IL1β* and *iNOS*) in macrophages in a dose- and time-dependent manner, while markedly suppressing the expression of anti-inflammatory genes (*CD206* and *Arg1*) (Fig. 3A, B). Consistently, the protein expression levels of the inflammatory mediators IL-1β, iNOS, and TNF-α exhibited similar trends (Fig. 3C). Flow cytometric analysis further revealed that SPP1 treatment markedly increased the surface expression of the co-stimulatory molecule CD86 and the proportion of TNF-α-positive macrophages, accompanied by a notable elevation in intracellular reactive oxygen species levels (Fig. 3D-I).

Furthermore, transmission electron microscopy revealed that SPP1-stimulated macrophages exhibited pronounced mitochondrial abnormalities, including disorganized cristae, swelling, and partial vacuolization, suggesting that SPP1 induces mitochondrial dysfunction—consistent with the elevated oxidative stress previously observed. In addition, SPP1 stimulation triggered the formation of autolysosome within macrophages (Fig. 3J). Moreover, wound-healing assays demonstrated that SPP1 promoted macrophage migration, with a greater migration distance observed after 48 h of stimulation compared to that with the control group (Fig. 3K). Collectively, these findings indicate that exogenous SPP1 not only drives macrophages toward a pro-inflammatory phenotype and enhances their migratory capacity but also elevates their oxidative stress levels, thereby contributing to the early phase of AKI.

### 3.4. SPP1 drives PKM2-mediated glycolytic metabolic reprogramming in macrophages

**Figure 4:**
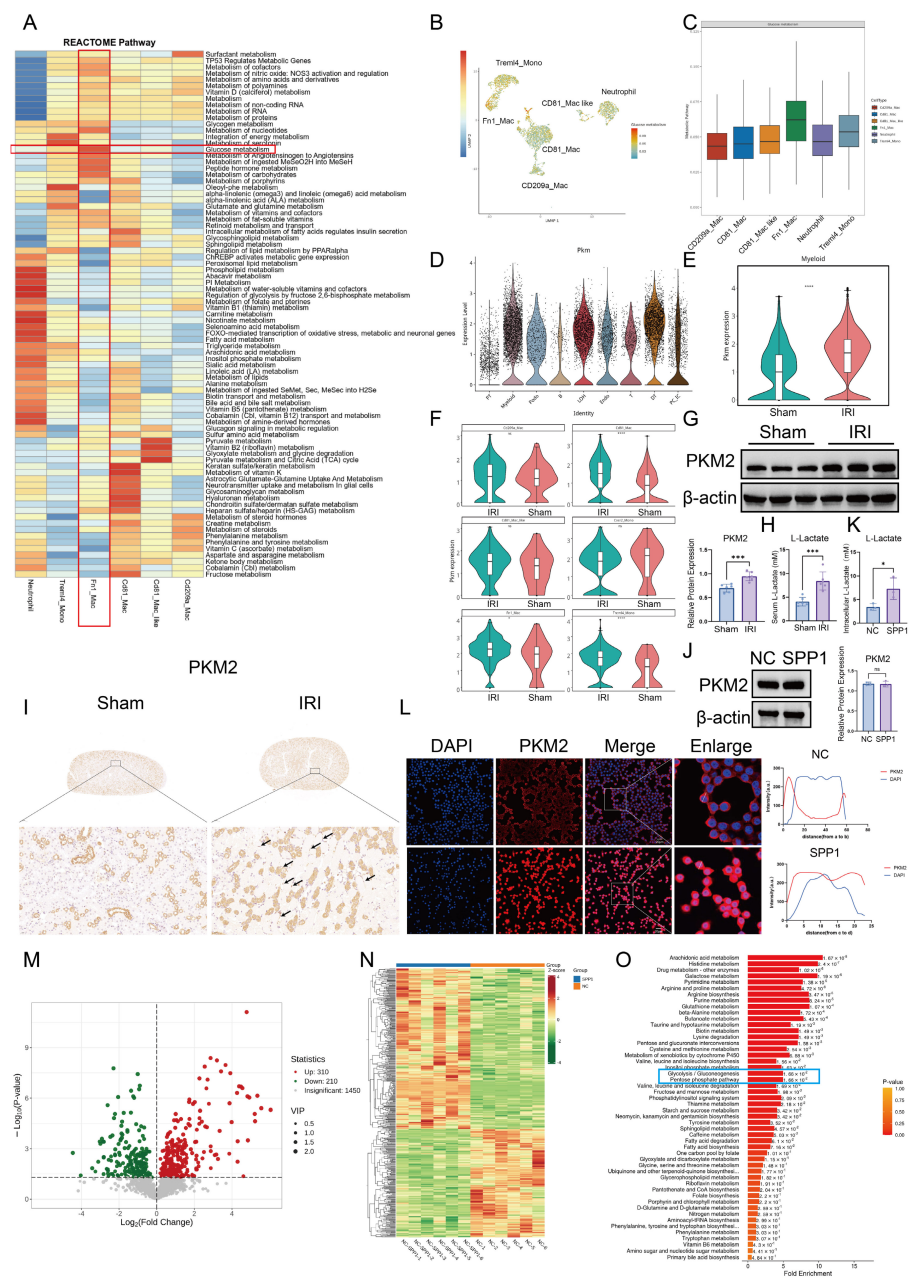
** SPP1 drives PKM2-mediated glycolytic metabolic reprogramming in macrophages.** (**A**) Reactome-based enrichment analysis of metabolic pathways in distinct immune cell subsets under the ischemia-reperfusion injury (IRI) microenvironment. (**B**) UMAP visualization of metabolic characteristics in different immune cell subsets. (**C**) Analysis of metabolic pathway activity in each immune cell subset. (**D**) Cellular origin analysis of PKM2 expression profile in IRI kidneys. (**E**) Comparison of PKM2 transcriptional levels in myeloid cells between the Sham and IRI groups. (**F**) Analysis of PKM2 expression across distinct myeloid cell subsets. (**G**) PKM2 protein expression and semi-quantitative analysis in renal tissues of the Sham and IRI groups. (**H**) Serum lactate levels in mice from the Sham and IRI groups. (**I**) Immunohistochemical staining of PKM2 in renal interstitial areas with inflammatory cell infiltration in the Sham and IRI groups. (**J**) PKM2 protein expression and semi-quantitative analysis in macrophages upon SPP1 stimulation. (**K**) Quantitative analysis of intracellular lactate levels in macrophages upon SPP1 stimulation. (**L**) Immunofluorescence analysis of PKM2 nuclear translocation in macrophages upon SPP1 stimulation. (**M**) Statistical analysis of the number of differential metabolites in macrophages following SPP1 stimulation. (**N**) Heatmap of metabolic profiles in macrophages between SPP1-treated and control groups. (**O**) Kyoto Encyclopedia of Genes and Genomes (KEGG) pathway enrichment analysis of metabolic changes in macrophages upon SPP1 stimulation.

To further elucidate the mechanism by which injured PT cells establish functional crosstalk with macrophages through the SPP1-CD44 signaling axis, we analyzed selectively activated pathways in immune cell populations based on previous single-cell transcriptomic data. Reactome pathway enrichment analysis revealed that, within the IRI microenvironment, sugar metabolism and carbohydrate metabolism pathways were broadly and significantly activated in Fn1⁺ macrophages, indicating pronounced metabolic reprogramming (Fig. 4A). We next visualized glycolytic metabolic activity across different myeloid cell subsets using Uniform Manifold Approximation and Projection (UMAP) plots and bar charts. Fn1⁺ macrophages were identified as one of the primary myeloid populations undergoing glycolytic metabolic reprogramming (Fig. 4B, C). Subsequently, we focused on the expression profile of PKM, a key rate-limiting enzyme in glycolysis. Elevated PKM signal in IRI kidneys originated predominantly from myeloid cells (Fig. 4D). At the subpopulation level, compared with that in the Sham group, overall transcription of *PKM* in myeloid cells was significantly increased in the IRI group (Fig. 4E), with the most pronounced gene expression observed in CD81⁺ macrophages, Treml4⁺ monocytes, and Fn1⁺ macrophages (Fig. 4F). Consistently, the total protein level of PKM2 in renal tissue was significantly higher in the IRI group than that in the Sham group (Fig. 4G). Furthermore, the serum concentration of lactate, a terminal product of glycolysis, was elevated in IRI mice compared to that in the Sham mice, providing additional evidence of enhanced glycolysis post-IRI (Fig. 4H). To confirm PKM2 protein expression in macrophages in situ, immunohistochemical staining was performed, which showed high PKM2 expression in macrophages located within inflammatory infiltrates in IRI kidneys (Fig. 4I). *In vitro* stimulation of RAW264.7 macrophages with recombinant SPP1 protein did not alter total PKM2 protein levels (Fig. 4J). However, intracellular lactate levels were significantly elevated (Fig. 4K), indicating that SPP1 stimulation enhances glycolytic activity in macrophages. Furthermore, immunofluorescence analysis revealed that SPP1 stimulation promoted nuclear accumulation of PKM2 (Fig. 4L), suggesting that nuclear translocation of PKM2 dimers is a key event in SPP1-mediated regulation of macrophage metabolism and inflammatory responses.

To systematically investigate the impact of SPP1 on macrophage metabolism, untargeted metabolomic analysis was performed on macrophages following SPP1 stimulation. A total of 520 metabolites were significantly altered compared to those in the control group, including 310 upregulated and 210 downregulated (Fig. 4M). A heatmap of differential metabolites clearly distinguished the distinct metabolic profiles between the SPP1-treated and control groups (Fig. 4N). Further KEGG pathway enrichment analysis was conducted to interpret the functional implications of these changes. Glycolysis/gluconeogenesis and the pentose phosphate pathway were significantly enriched (Fig. 4O), with levels of key metabolites within these pathways consistently elevated, including xylitol, sedoheptulose, DL-glyceraldehyde-3-phosphate, D-Glycero-D-gulo-heptose, mannose, pentitol, glucose, D-Glucaro-1,4-lactone and threitol (Fig. S3). These findings suggest that SPP1 induces extensive metabolic reprogramming in macrophages, with activation of the glycolytic pathway being a prominent feature.

### 3.5. CD44 mediates SPP1-induced metabolic reprogramming and pro-inflammatory polarization of macrophages

**Figure 5:**
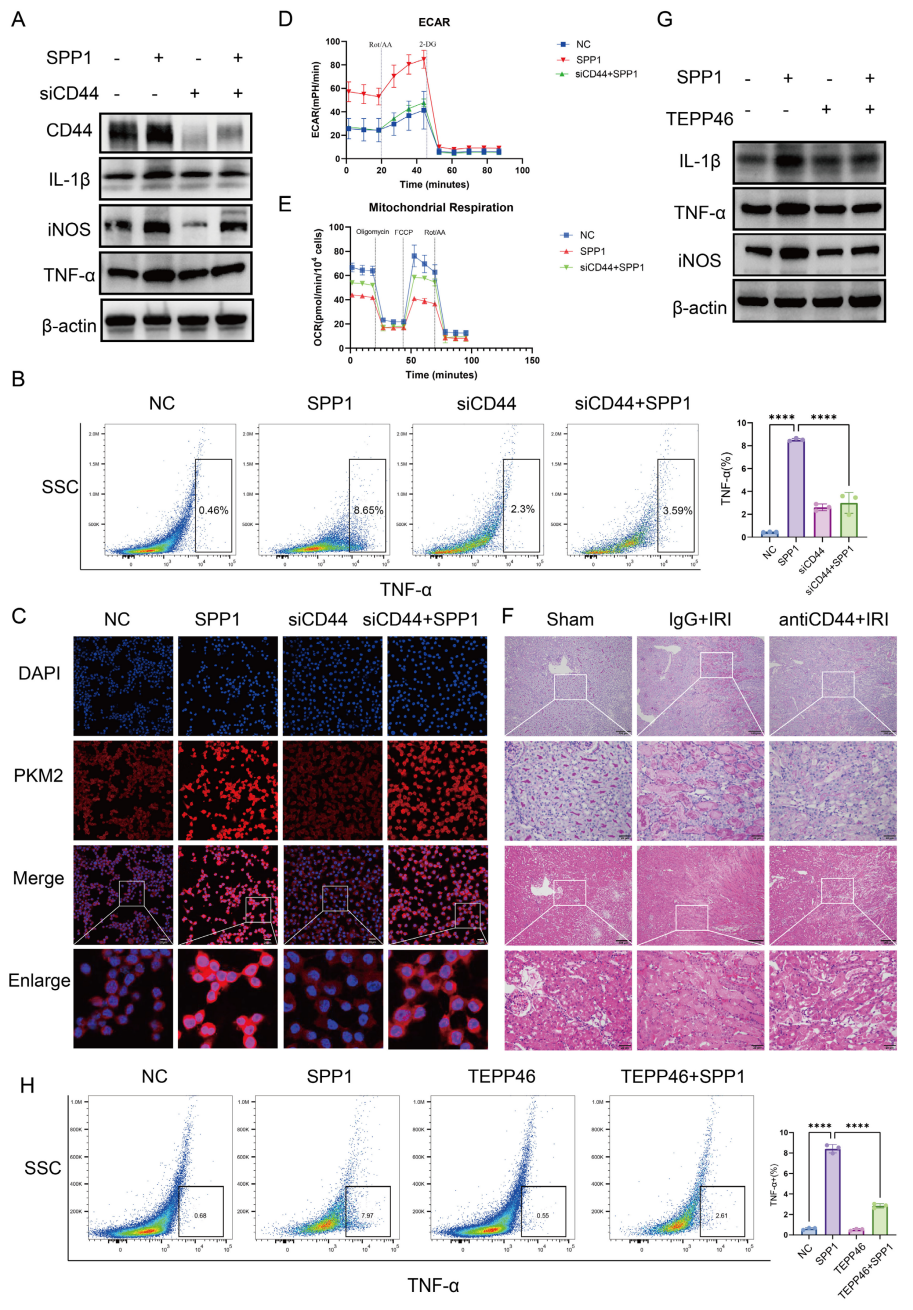
** CD44 mediates SPP1-induced metabolic reprogramming and pro-inflammatory polarization of macrophages.** (**A**) Effects of siRNA-mediated CD44 knockdown on SPP1-induced pro-inflammatory protein expression in macrophages, evaluated via western blotting. (**B**) Effects of CD44 knockdown on SPP1-induced tumor necrosis factor alpha (TNF-α) expression in macrophages, examined via flow cytometry. (**C**) Effects of CD44 knockdown on SPP1-induced PKM2 nuclear translocation in macrophages, assessed via immunofluorescence staining. (**D** and **E**) Effects of CD44 knockdown on SPP1-induced macrophage glycolysis (extracellular acidification rate, ECAR) and mitochondrial respiration (oxygen consumption rate, OCR), measured using the Seahorse assay. (**F**) Effects of CD44 neutralizing antibody treatment on renal damage in IRI mice, assessed using hematoxylin and eosin (H&E) and periodic acid-Schiff (PAS) staining. (**G**) Inhibitory effects of TEPP-46 on SPP1-induced upregulation of pro-inflammatory protein expression in macrophages. (**H**) Inhibitory effects of TEPP-46 on SPP1-induced TNF-α expression in macrophages, detected using flow cytometry.

To determine whether CD44 mediates SPP1-induced pro-inflammatory activation and metabolic reprogramming in macrophages, we established a stable CD44 knockdown model in RAW264.7 cells using siRNA. The knockdown efficiency reached approximately 80% at both the mRNA and protein levels (Fig. 5A; S4A-B).

CD44 knockdown effectively antagonized the pro-inflammatory effects of SPP1. At the gene expression level, SPP1-induced upregulation of pro-inflammatory genes (*IL1β* and *iNOS*) was significantly inhibited, while expression of anti-inflammatory *CD206* was partially restored (Fig. S4A). Western blot analysis validated this trend, showing that, following CD44 knockdown, SPP1-induced protein expression of IL-1β, iNOS, and TNF-α was significantly reduced (Fig. 5A; S4B). Flow cytometry also verified a decrease in the proportion of TNF-α-positive cells (Fig. 5B).

At the metabolic level, CD44 knockdown similarly reversed the SPP1-induced change of the key glycolytic enzyme PKM2 (Fig. 5C). Seahorse metabolic analysis further showed that SPP1 stimulation significantly enhanced the glycolytic capacity of macrophages, as indicated by increased extracellular acidification rate, with substantial elevations in basal glycolysis, compensatory glycolytic capacity, and glycolytic ATP production. CD44 knockdown partially reversed these increases (Fig. 5D; Fig. S4C, S4D). Concurrently, the SPP1-induced suppression of Oxygen Consumption Rate—including reductions in basal respiration, maximal respiration, spare respiratory capacity, and proton leak—was partially restored following CD44 knockdown (Fig. 5E; Fig. S4E).

Furthermore, flow cytometry-based cell cycle analysis revealed that SPP1 stimulation promoted macrophage entry into the S phase. This effect was reversed upon CD44 knockdown (Fig. S4F), suggesting that the enhanced glycolysis driven by the SPP1-CD44 axis may support cell proliferation at both energetic and biosynthetic levels.

In a mouse model of renal IRI, treatment with a CD44 neutralizing antibody was performed. Compared with the IgG control group, mice receiving anti-CD44 exhibited significantly attenuated renal injury, as evidenced by reduced tubular necrosis and cast formation in both PAS and H&E staining (Fig. 5F). Concurrently, levels of Scr and BUN were markedly lower in the anti-CD44 group (Fig. S4G). At the molecular level, western blot analysis showed that the protein expression of the injury marker Kim-1, as well as the inflammatory factors IL-1β and iNOS, was decreased in renal tissue of the anti-CD44 group compared to those in the IRI group (Fig. S4H). Collectively, these findings indicate that CD44 mediates SPP1-induced pro-inflammatory polarization and metabolic reprogramming of macrophages.

To confirm that SPP1-induced nuclear translocation of PKM2 in macrophages drives pro-inflammatory activation rather than being merely correlative, we employed TEPP-46, a specific PKM2 activator, which promotes the formation of highly active PKM2 tetramers, thereby competitively inhibiting the nuclear translocation of its dimeric form and subsequent function as a transcriptional co-activator. Immunofluorescence analysis showed that, compared to SPP1 treatment alone, TEPP-46 pretreatment effectively blocked SPP1-driven nuclear accumulation of PKM2 (Fig. S5A). Building on this, TEPP-46 treatment significantly reversed the pro-inflammatory effects of SPP1. At the transcription level, the SPP1-induced upregulation of pro-inflammatory cytokine mRNAs (*IL1β*, *iNOS*, *IL6*, *TNF-α*) was markedly suppressed (Fig. S5B). This trend was also confirmed at the protein level, with western blot analysis showing a concurrent decrease in the protein expression of IL-1β, iNOS, and TNF-α (Fig. 5G; S5C), and flow cytometric analysis revealing a significant reduction in the proportion of TNF-α-positive cells (Fig. 5H). These results demonstrate that pharmacologically inhibiting PKM2 nuclear translocation attenuates SPP1-induced pro-inflammatory responses in macrophages. Collectively, these findings indicate that SPP1 regulates macrophage pro-inflammatory polarization by driving the nuclear accumulation of PKM2 dimers.

### 3.6. SPP1-CD44 axis drives PKM2 nuclear translocation and macrophage pro-inflammatory polarization through PI3K/AKT pathway activation

**Figure 6:**
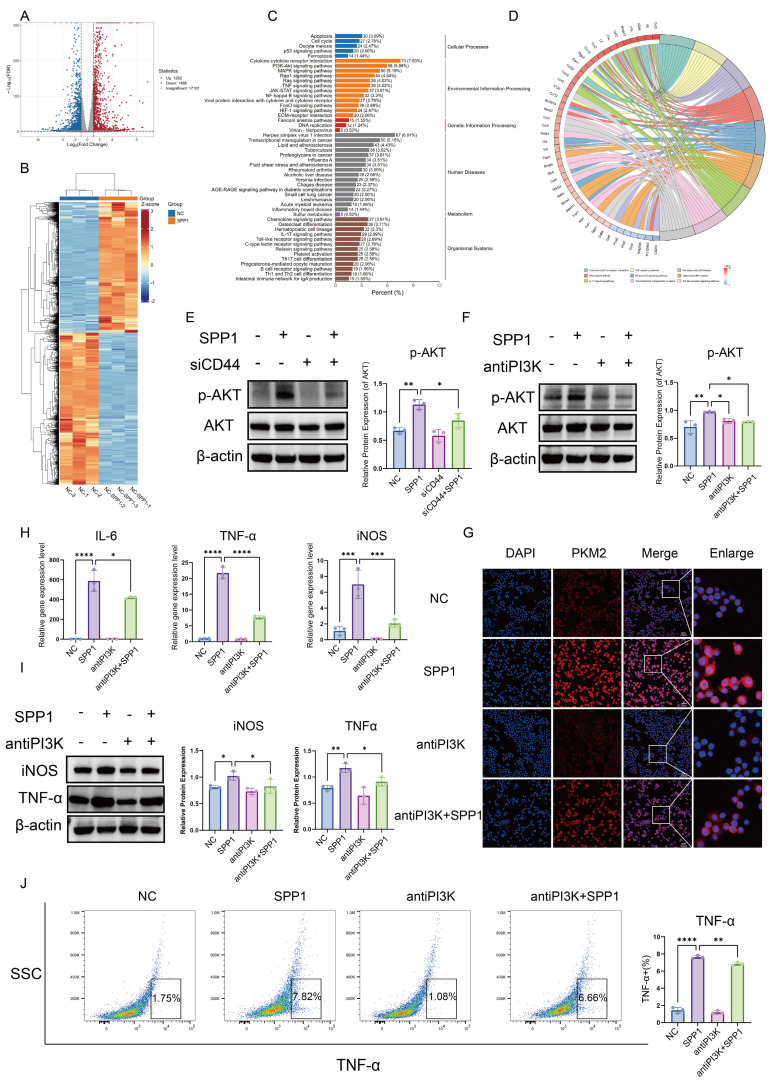
** The SPP1-CD44 axis drives PKM2 nuclear translocation and macrophage pro-inflammatory polarization through PI3K/AKT pathway activation.** (**A**) Number of differentially expressed genes in macrophages following SPP1 stimulation. (**B**) Heatmap of transcriptional profile differences in macrophages between SPP1-stimulated and control groups. (**C**) Kyoto Encyclopedia of Genes and Genomes (KEGG) pathway enrichment analysis of genes differentially expressed upon SPP1 stimulation. (**D**) Chord diagram showing correlations among SPP1-induced key inflammatory factors and chemokines. (**E**) Activation of the PI3K/AKT pathway at the protein level in macrophages upon SPP1 stimulation. (**F**) Validation of the suppressive effect of a PI3K inhibitor on SPP1-induced AKT phosphorylation at the protein level. (**G**) Analysis of the suppressive effect of the PI3K inhibitor on SPP1-induced PKM2 nuclear translocation via immunofluorescence staining. (**H**) Suppressive effect of the PI3K inhibitor on SPP1-induced upregulation of pro-inflammatory factor gene expression detected via quantitative polymerase chain reaction (qPCR). (**I**) Validation of the suppressive effect of the PI3K inhibitor on SPP1-induced upregulation of pro-inflammatory protein expression. (**J**) Suppressive effect of the PI3K inhibitor on SPP1-induced TNF-α expression in macrophages, evaluated using flow cytometry.

To elucidate how the SPP1-CD44 signal is transduced intracellularly to regulate PKM2 nuclear translocation, transcriptomic sequencing analysis was performed on macrophages following SPP1 stimulation. A total of 2,730 genes with significantly altered expression were identified, including 1,262 upregulated and 1,468 downregulated genes (Fig. 6A). A heatmap of gene expression illustrated the distinct transcriptional profiles between the SPP1-stimulated and control groups (Fig. 6B).

KEGG pathway enrichment analysis indicated that the PI3K/AKT signaling pathway was among the most significantly enriched activated pathways (Fig. 6C). Furthermore, circos plot analysis of pathway gene associations revealed that the activation of this pathway was closely linked to alterations in the expression of multiple inflammation-related genes. Notably, SPP1 stimulation significantly upregulated the expression of pro-inflammatory cytokines secreted by macrophages, including IL-6, IL-1β, and TNF-α, as well as several chemokines, such as CCL2, CXCL3, CCL22, and CXCL2 (Fig. 6D), which is highly consistent with our previous findings.

Based on these findings, the activating effects of SPP1 on the PI3K/AKT pathway at the protein level were evaluated. Western blot analysis showed that SPP1 stimulation significantly increased the level of phosphorylated AKT (p-AKT), whereas the total amount of AKT protein remained largely unchanged (Fig. 6E). Furthermore, a specific PI3K inhibitor effectively suppressed SPP1-induced AKT phosphorylation (Fig. 6F), indicating successful blockade of the PI3K/AKT signaling pathway.

Blocking the PI3K/AKT signal substantially reversed SPP1-driven nuclear accumulation of PKM2 (Fig. 6G). A consistent trend was observed for inflammatory mediators: at the mRNA level, SPP1-induced upregulation of TNF-α, iNOS, and IL-6 expression was markedly inhibited (Fig. 6H), and at the protein level, western blot and flow cytometric analyses showed downregulation of iNOS and TNF-α levels (Fig. 6I, J). Collectively, these findings demonstrate that the PI3K/AKT signaling pathway serves as a central link connecting the extracellular SPP1-CD44 signal to intracellular PKM2 nuclear translocation. By regulating both the expression and subcellular localization of PKM2, this pathway mediates the pro-inflammatory polarization of macrophages.

### 3.7. Targeting or genetically ablating tubular SPP1 attenuates renal IRI

**Figure 7:**
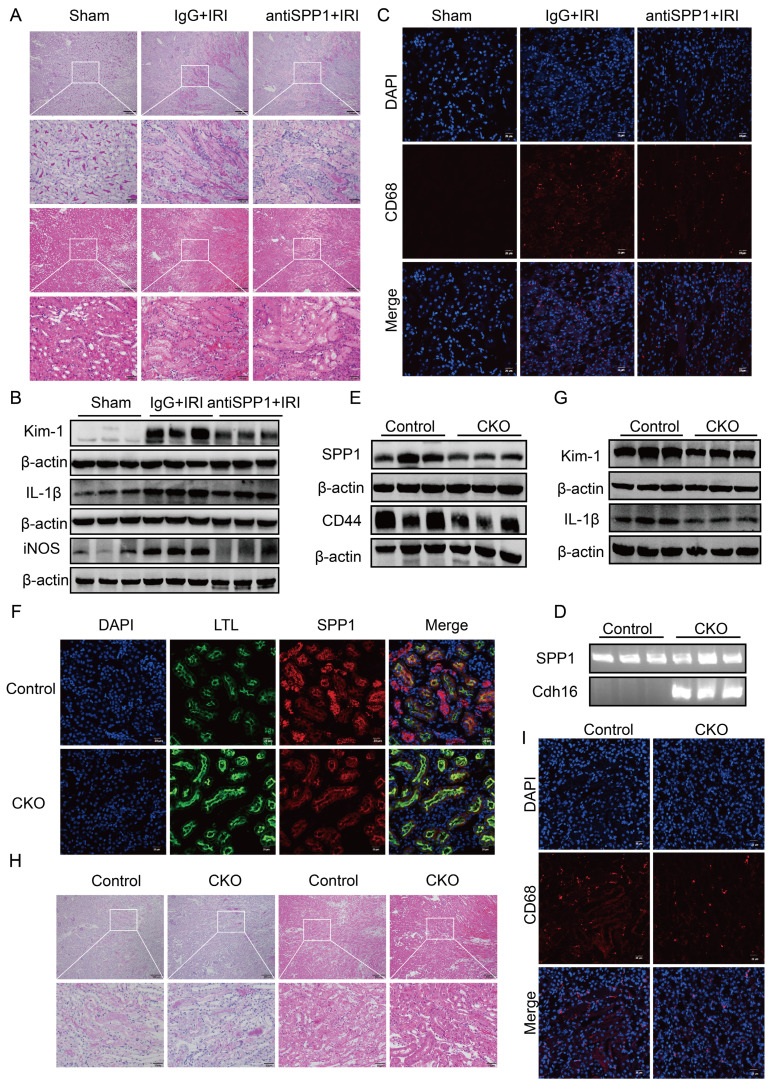
** Targeting or genetically ablating tubular SPP1 attenuates renal IRI.** (**A**) Effects of SPP1 neutralizing antibody treatment on renal damage in IRI mice, assessed via H&E and PAS staining. (**B**) Effects of the SPP1 neutralizing antibody on renal injury and inflammatory factor protein expression in IRI mice. (**C**) Effects of the SPP1 neutralizing antibody on renal macrophage infiltration in IRI mice assessed via immunofluorescence staining. (**D**) Genotyping of tail DNA from renal tubular epithelial cell-specific SPP1 conditional knockout mice (*Spp1*^flox/flox^; *Cdh16*-Cre). (**E**) Knockout efficiency of renal tubular epithelial cell-specific SPP1 deletion and associated renal CD44 expression. (**F**) *In vivo* validation of renal tubular epithelial cell-specific SPP1 knockout, evaluated using immunofluorescence staining. (**G**) Effects of SPP1 conditional knockout on renal injury and inflammation-related protein expression in IRI mice. (**H**) Effects of SPP1 conditional knockout on renal damage in IRI mice, assessed via H&E and PAS staining. (**I**) Effects of SPP1 conditional knockout on renal macrophage infiltration in IRI mice, examined using immunofluorescence staining.

To evaluate the therapeutic potential of targeting SPP1 in renal IRI *in vivo*, we first employed a neutralizing antibody approach. Compared to the IgG-treated IRI control group, mice receiving the anti-SPP1 antibody exhibited markedly attenuated renal injury, as evidenced by substantially reduced tubular necrosis, cast formation, and structural disruption upon both PAS and H&E staining (Fig. 7A). Consistent with this, Scr and BUN levels were lower in the treatment group compared to those in the controls (Fig. S6A), indicating notable improvement in renal function.

At the protein level, western blot analysis revealed that the expression levels of the renal injury marker Kim-1 and pro-inflammatory cytokine IL-1β in renal tissue were markedly lower in the anti-SPP1 treatment group compared to those in the IRI control group (Fig. 7B; Fig. S6B). Immunofluorescence staining further confirmed that anti-SPP1 treatment led to a marked reduction in CD68^+^ signals within the renal tissue (Fig. 7C), indicating suppressed macrophage infiltration.

At the genetic level, a renal tubular epithelial cell-specific SPP1 conditional knockout mouse model was established. Genotyping confirmed *Spp1*^flox/flox^; *Cdh16*-Cre^-^ mice as the control group and *Spp1*^flox/flox^; *Cdh16*-Cre⁺ mice as the experimental group (Fig. 7D). Western blot analysis confirmed a reduction of approximately 50% in SPP1 expression in the kidneys of the experimental group following IRI (Fig. 7E; Fig. S6C). Immunofluorescence staining further validated the specific knockout of SPP1 in renal tubular epithelial cells at the tissue level (Fig. 7F).

Compared with control mice, SPP1 conditional knockout mice exhibited significantly attenuated renal damage (Fig. 7H) and markedly lower Scr and BUN levels (Fig. S6D) after IRI. Western blot analysis showed that the expression of Kim-1, IL-1β, and CD44 in kidney tissues was notably downregulated in the knockout group compared to that in the control group (Fig. 7G, 7E; Fig. S6E). Immunofluorescence staining further demonstrated a marked reduction in CD68⁺ macrophage infiltration within the renal tissue upon SPP1 knockout (Fig. 7I).

Notably, serum lactate levels were also decreased in SPP1 conditional knockout mice compared to those in the control group after IRI (Fig. S6F), indicating that SPP1-mediated glycolytic activation was attenuated during the injury process *in vivo*.

Taken together, these findings demonstrate that both systemic SPP1 blockade and renal tubular epithelial cell-specific SPP1 knockout effectively mitigate IRI-induced renal pathological damage, improve renal function, and suppress inflammatory cell infiltration, and highlight a key pathogenic role for SPP1 in renal IRI *in vivo*.

## 4. Discussion

This study integrates scRNA-seq and metabolomic data with gene knockout technology and functional validation experiments to systematically elucidate the key mechanisms underlying early inflammatory progression in renal IRI by examining intercellular communication. Specifically, injured renal PT epithelial cells secrete SPP1, which binds to CD44 on the surface of macrophages, thereby activating the downstream PI3K/AKT signaling pathway and inducing nuclear translocation of PKM2; this cascade coordinately regulates glycolytic metabolic reprogramming and pro-inflammatory phenotypic transformation in macrophages. To the best of our knowledge, this study is the first to integrate tissue damage signals, immunometabolic reprogramming, and inflammatory outcomes at the single-cell and metabolic levels, providing new insights on the pathological mechanisms of AKI.

First, this study is the first to identify a novel intercellular communication mechanism mediated by the SPP1-CD44 axis in renal IRI-induced AKI at single-cell resolution using scRNA-seq technology. Consistent with the findings in ischemic stroke models, SPP1 is involved in neutrophil- and monocyte-mediated inflammation, whereas inhibition of SPP1 significantly reduces the infiltration of these inflammatory cells and attenuates the inflammatory response 10. Additionally, other studies have demonstrated that SPP1 is a key mediator of kidney-lung crosstalk after AKI. Specifically, renal PT epithelial cells secrete SPP1, which triggers pulmonary endothelial barrier disruption, inflammation, and respiratory failure; in contrast, pharmacological or genetic inhibition of SPP prevents AKI-induced acute lung injury 11. Subsequently, this study focused on tracing the cellular sources of SPP1, revealing that different tubular segments, including the PT, LOH, and DT, all secrete SPP1 within the AKI tissue microenvironment. Based on the histopathological finding that the most severely injured PT cells at the corticomedullary junction colocalize with macrophages, we focused on the role of SPP1 secreted by these injured PT cells. SPP1 acts on pro-inflammatory myeloid cell subsets, including Treml4⁺ monocytes and Fn1⁺ macrophages, that highly express CD44, promoting their acquisition of a pro-inflammatory phenotype. This observation is consistent with the findings of Salathia *et al*., which identified CD44 as a marker for pro-inflammatory macrophage activation 12. This research paradigm overcomes the limitations of previous bulk tissue studies, precisely delineates the interaction pattern between injured PT cells and pro-inflammatory macrophages, and demonstrates the unique advantages of single-cell resolution. However, the spatial localization of tissues is still unknown, highlighting a direction of future research.

Next, this study further investigates the core mechanisms by which the SPP1-CD44 axis drives the pro-inflammatory phenotypic transformation of macrophages. Consistent with the scRNA-seq results, Seahorse metabolic assays and metabolomics analysis indicated that macrophages stimulated with exogenous recombinant SPP1 undergo glycolysis-dominant metabolic reprogramming. This enhanced glycolysis not only meets the high energy demands for cell activation, migration, and synthesis of inflammatory mediators through rapid ATP supply, but also provides substrates for nucleotide synthesis and NADPH production via the pentose phosphate pathway branch. NADPH is essential for lipid synthesis and serves as a cofactor for NADPH oxidase in generating ROS, which, as signaling molecules, further amplify the inflammatory response. These findings align with the well-established concept that pro-inflammatory polarization is closely associated with enhanced glycolysis 13-15. Moreover, following SPP1 gene knockout or blockade of the macrophage CD44 receptor, macrophage infiltration is reduced, inflammatory responses are attenuated, and renal function is partially restored in AKI. Collectively, these findings confirm that the SPP1-CD44 axis is a key upstream signaling pathway driving this metabolic switch. Furthermore, this study employed bulk RNA-seq and metabolomics to dissect the signaling pathways by which SPP1 activates pro-inflammatory macrophages. The PI3K/AKT pathway was among the two most significantly activated signaling pathways, accompanied by the nuclear translocation of PKM2, without changes in its total protein expression. This finding is consistent with the report by Park *et al*., which demonstrated that the PI3K/AKT pathway regulates the nuclear localization of PKM2 16. However, this study did not investigate other factors influencing the enzymatic activity of PKM2, such as lactylation modifications. In summary, this study elucidates a cascade mechanism of metabolic reprogramming mediated by PKM2 nuclear translocation of the SPP1-CD44 axis, linking tissue damage signals, immune cell metabolism, and inflammatory responses, and provides systematic mechanistic insights into the formation of the immunometabolic microenvironment in AKI.

This study demonstrates that the SPP1-CD44 axis mediates the pro-inflammatory phenotypic transformation of macrophages by activating the PI3K/Akt signaling pathway and regulating PKM2 nuclear translocation. However, the study has several limitations that warrant further investigation: (i) This study primarily focused on the regulation of macrophage metabolic reprogramming and phenotypic transformation by the SPP1-CD44 axis via the PI3K/Akt signaling pathway. However, whether this axis initiates or interacts with other key signaling pathways, such as MAPK, NF-κB and JAK-STAT, within the complex *in vivo* microenvironment, thereby synergistically amplifying the inflammatory response, remains unclear. (ii) In macrophages, existing studies have confirmed that PKM2 can interact with factors such as STAT3, HIF-1α, and NF-κB p65 to cooperatively regulate the expression of specific pro-inflammatory genes, including IL-1β 17-21. Nevertheless, in the context of renal IRI, the mechanisms by which the PI3K/Akt signaling pathway precisely regulates PKM2 dimerization, nuclear translocation, and its specific transcriptional partners and target genes remain to be elucidated. A precise regulatory map of PKM2 in kidney-infiltrating inflammatory macrophages should be delineated using techniques such as ChIP-seq. (iii) Whether SPP1 derived from different phases and cellular sources—such as early injured, later reparative, and fibrogenic PT cells, or via macrophage autocrine signaling—exhibits intrinsic functional differences, and the mechanisms underlying these functional shifts, represent key directions for future research. (iv) From a translational medicine perspective, our *in vivo* experiments have demonstrated that targeting either SPP1 or CD44 effectively alleviates renal IRI in mice, consistent with previous research findings 22. However, given that SPP1 also plays critical roles in physiological processes such as bone metabolism, translating this strategy of inhibiting SPP1 to alleviate AKI into clinical practice requires careful consideration 23, 24. Future studies should explore more targeted strategies, such as kidney-specific drug delivery or intervention at downstream signaling events, to maximize therapeutic efficacy while minimizing side effects.

In conclusion, this study is the first to comprehensively integrate scRNA-seq, untargeted metabolomics, and gene editing techniques. It systematically elucidates the mechanism by which severely injured PT cells in the early stage of AKI target macrophages and drive their metabolic reprogramming and pro-inflammatory polarization via the SPP1-CD44-PI3K/AKT-PKM2 signaling axis at the cellular, ligand-receptor, and metabolic molecular levels. This work provides potential therapeutic targets and a theoretical framework for the prevention and treatment of AKI and its progression to chronic kidney disease.

## Supplementary Material

Supplementary figures and table.

## Data Availability

Data are available upon request.

## Abbreviations

AKI: acute kidney injury
BUN: blood urea nitrogen
H&E: hematoxylin and eosin
IRI: ischemia-reperfusion injury
KEGG: Kyoto Encyclopedia of Genes and Genomes
PAS: periodic acid-Schiff
scRNA-seq: single-cell RNA sequencing
Scr: serum creatinine
SPP1: secreted phosphoprotein
