# Supplementary material for: The SPP1-CD44 Signaling Axis Orchestrates Macrophage Metabolism to Promote Early Inflammation in Acute Kidney Injury
Source: Int J Biol Sci. 2026 May 1;22(9):4900–18. doi: 10.7150/ijbs.130922 (PMC13182544; doi:10.7150/ijbs.130922)
Supplement: Supplementary file 1 — Supplementary figures and table. [file ijbsv22p4900s1.pdf]

SUPPLEMENTARY MATERIAL

Supplementary Table 1 Primer Sequences

Target Gene	Forward Primer Sequence (5′–3′)	Reverse Primer Sequence (5′–3′)
β-actin	CTTGGGTATGGAATCCTGTGG	AGGTCTTTACGGATGTCAACG
IL-1β	TCGTGCTGTCGGACCCATA	GTCGTTGCTTGGTTCTCCTTGT
IL-6	ATGAACAACGATGATGCACTTG	TACTCCAGAAGACCAGAGGAAA
TNF-α	GTTCTGTCCCTTTCACTCACTG	GGATCATGCTTTCTGTGCTCAT
iNOS	AGGAGAAGGGGACGAACTC	TGCATTGGAAGTGAAGCGT
CD206	TGGCTTATGGGATGTTTTGAGT	CATTTGGGTTCAGGAGTTGTTG
Arg1	CCTTGGCTTGCTTCGGAACTC	TGTCTGCTTTGCTGTGATGCC
CD44	TGGCTGTGTTTGTTGGTGCTTT	CCTGTGGCTTTTTGAGGGGTTC

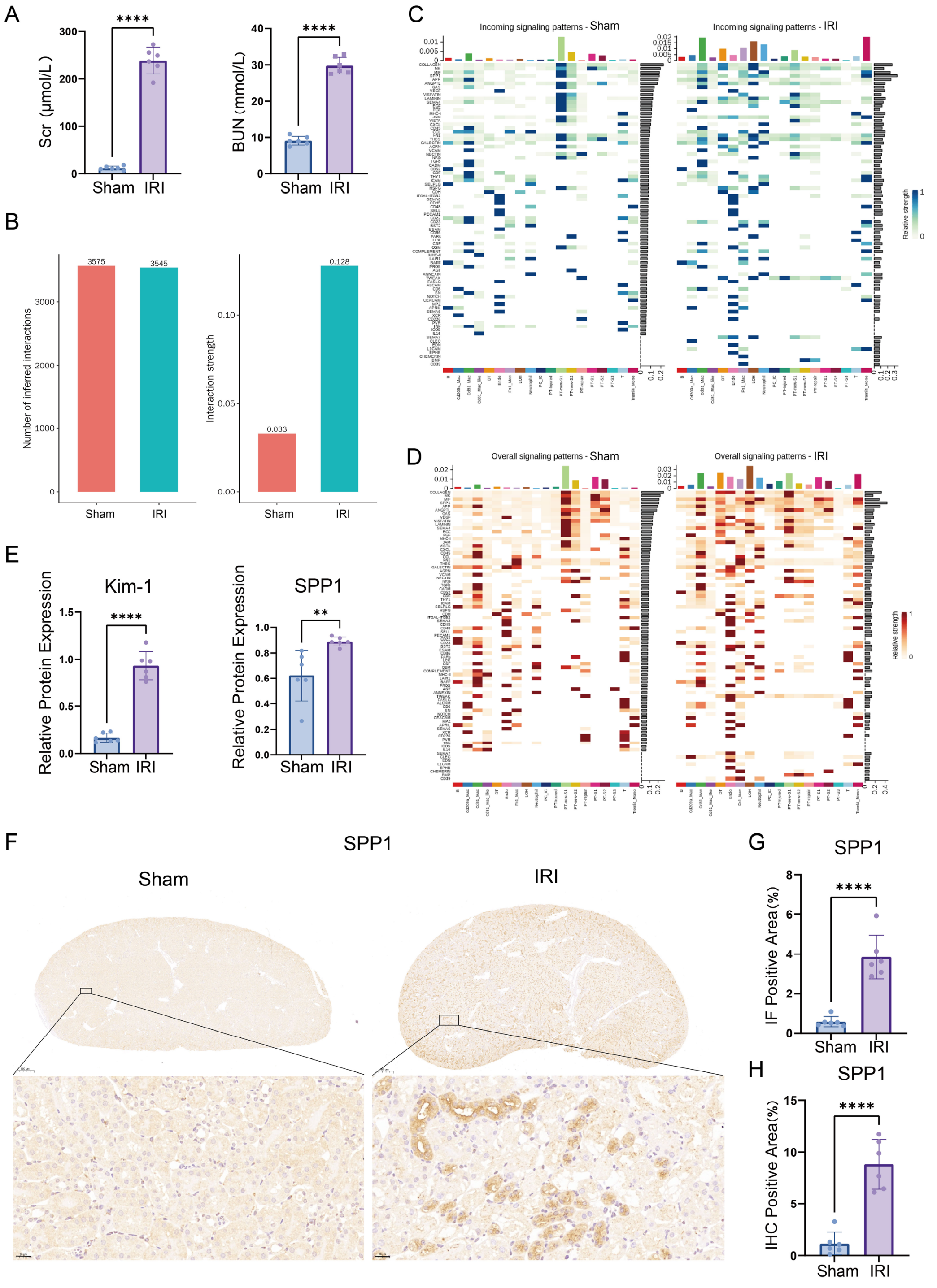

Fig. S1. The SPP1 pathway is involved in intercellular communication during IRI (**A**) Biochemical measurement of renal function indices (serum creatinine [Scr] and blood urea nitrogen [BUN]) in the Sham and IRI groups. (**B**) Comparison of the number of ligand–receptor interactions between the Sham and IRI groups. (**C**) Analysis of incoming signaling across different cell subpopulations in the Sham and IRI groups. (**D**) Analysis of overall signaling

activation in cell subpopulations from the Sham and IRI groups. (**E**) Semi-quantitative analysis of Kim-1 and SPP1 protein expression in the Sham and IRI groups. (**F**) Immunohistochemical staining of SPP1 in the Sham and IRI groups. (**G** and **H**) Semi-quantitative analysis of SPP1 immunohistochemistry and immunofluorescence.

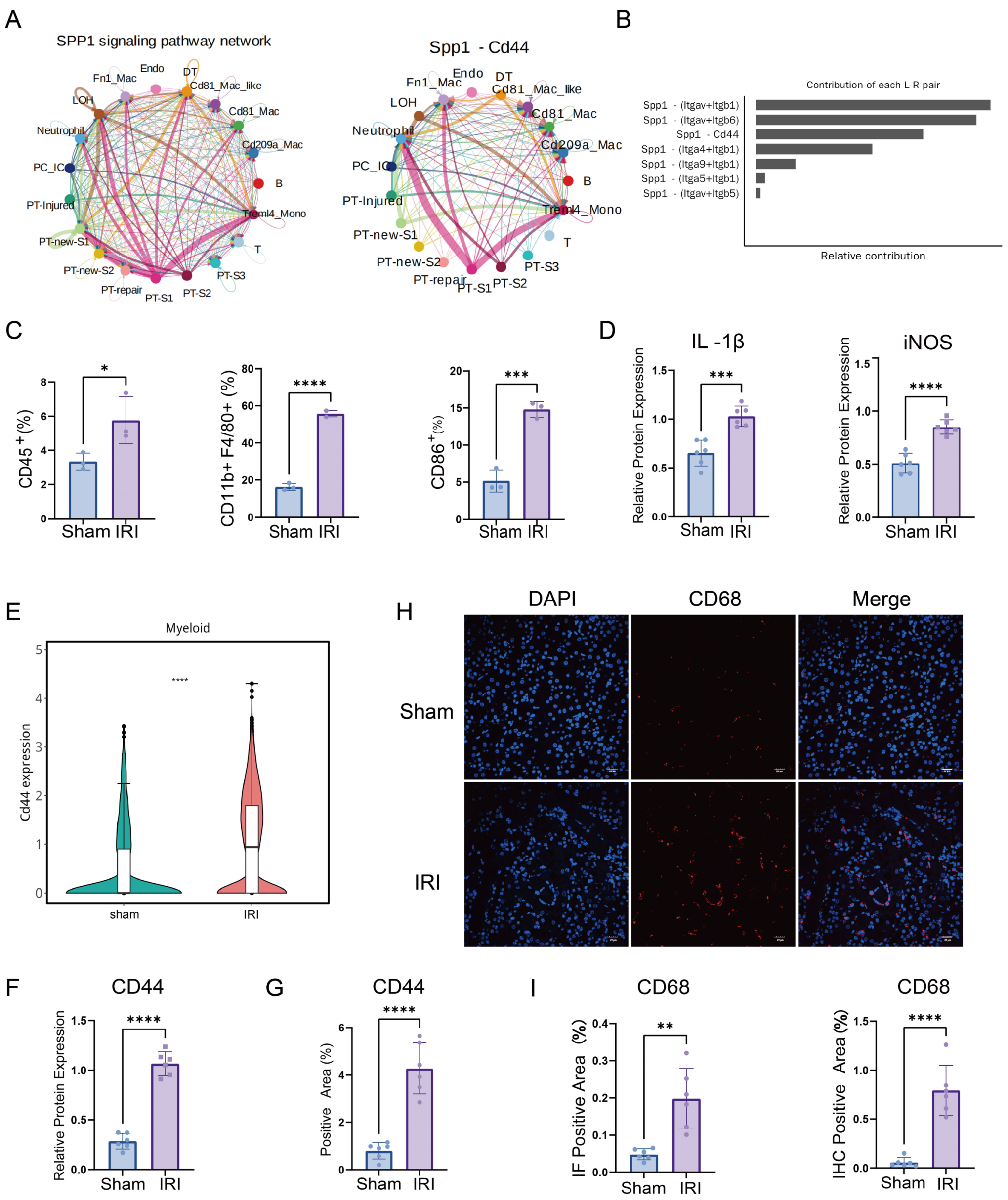

Fig. S2. The SPP1−CD44 axis mediates the interaction between injured PT cells and macrophages following IRI (**A** and **B**) SPP1 ligand-mediated intercellular interaction network and corresponding analysis. (**C**) Quantitative analysis of renal immune cell subset proportions in the Sham and IRI groups. (**D**) Semi-quantitative analysis of IL-1β and iNOS protein expression in the Sham and IRI groups. (**E**) Pan-expression profile of CD44 in myeloid cells based on single-cell RNA sequencing data. (**F**) Semi-quantitative analysis of renal CD44 protein expression using western blotting in the Sham and IRI groups. (**G**) Semi-quantitative analysis of renal CD44 expression via immunohistochemistry in the Sham and IRI groups. (**H**)

Immunofluorescence staining of renal macrophage infiltration in the Sham and IRI groups. (**I**) Semi-quantitative analysis of renal macrophage infiltration using immunofluorescence and immunohistochemistry in the Sham and IRI groups.

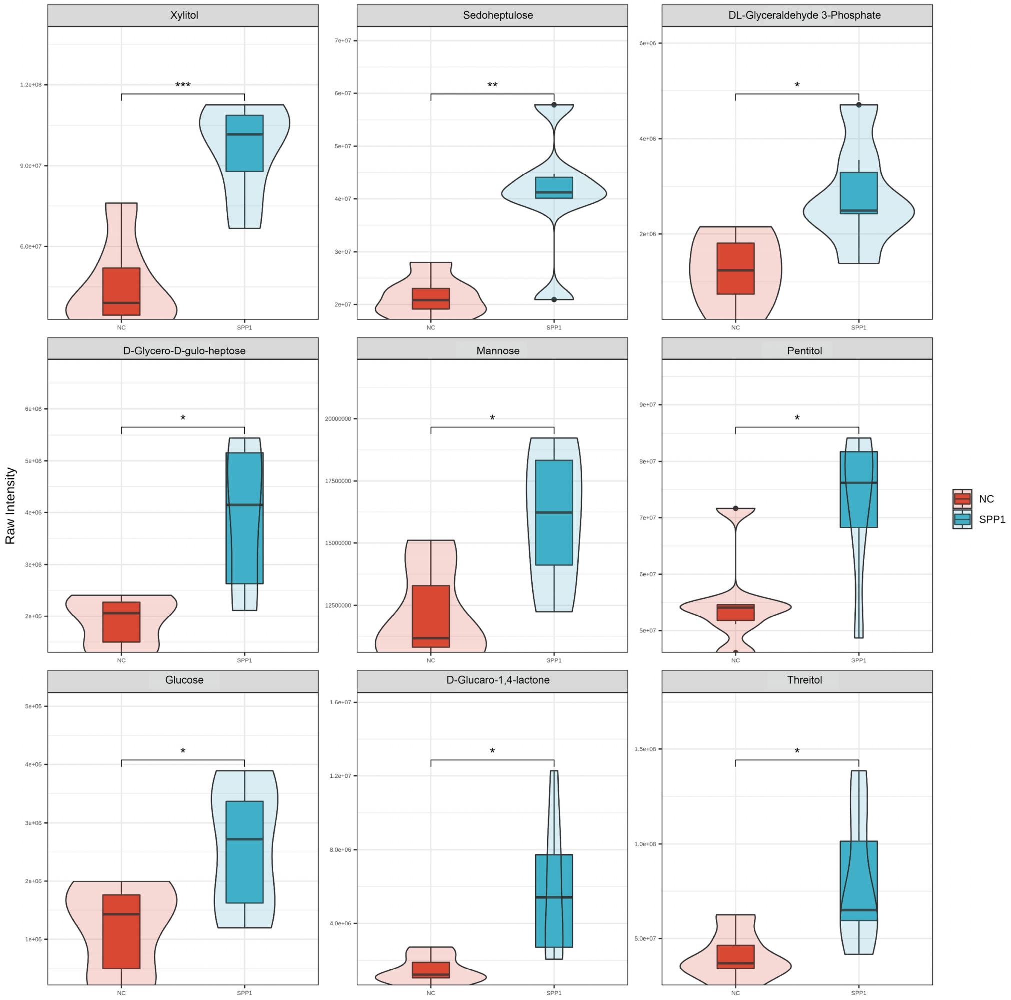

Figure S3. Visualization analysis of differential metabolites in carbohydrates in macrophages upon SPP1 stimulation.

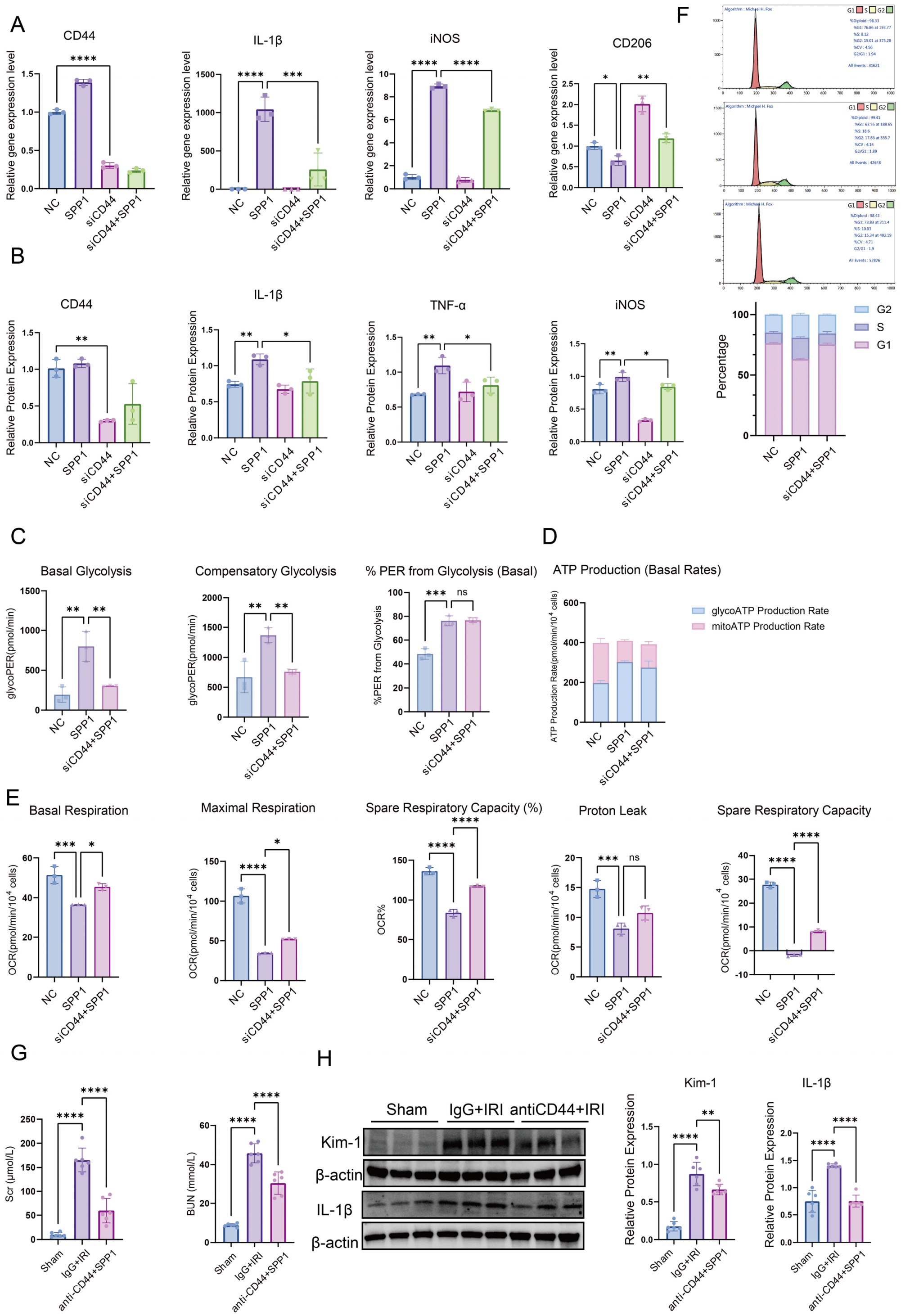

Figure S4. CD44 mediates SPP1-induced metabolic reprogramming and pro-inflammatory polarization of macrophages (**A**) Effects of CD44 knockdown on SPP1-induced pro-inflammatory gene expression, assessed via qPCR. (**B**) Semi-quantitative analysis of SPP1-induced pro-inflammatory protein expression following CD44 knockdown. (**C**–**E**)

Quantitative analysis of the effects of CD44 knockdown on SPP1-induced macrophage glycolysis (ECAR) and mitochondrial respiration (OCR). (**F**) Effects of CD44 knockdown on SPP1-induced macrophage cell cycle changes detected by flow cytometry. (**G**) Effects of CD44 neutralizing antibody treatment on renal function indices (Scr and BUN) in IRI mice. (**H**) Effects of CD44 neutralizing antibody treatment on renal injury and pro-inflammatory protein expression in IRI mice, with semi-quantitative analysis.

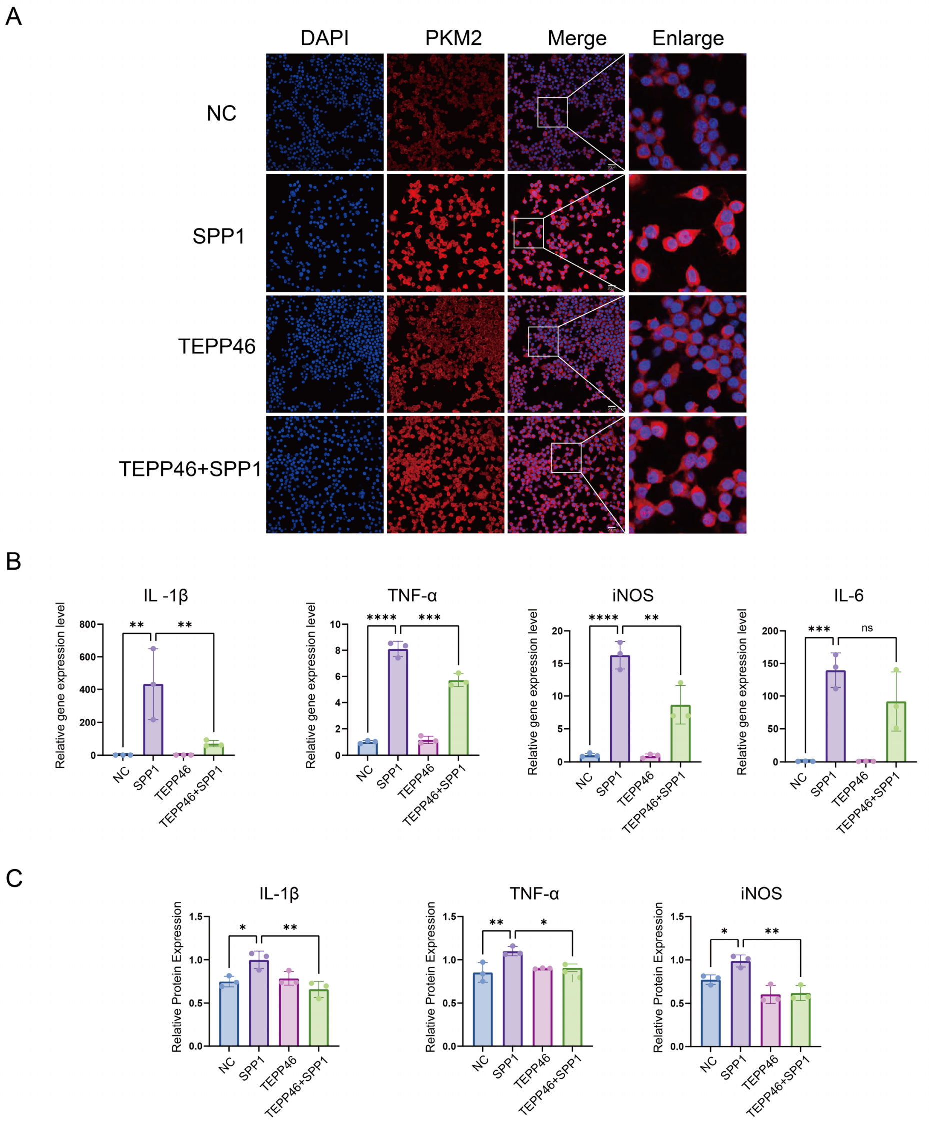

Figure S5. PKM2 nuclear translocation mediates SPP1-induced pro-inflammatory effects in macrophages (**A**) Immunofluorescence validation of the suppressive effect of PKM2 agonist TEPP-46 on SPP1-induced PKM2 nuclear translocation in macrophages. (**B**) Suppressive effect of TEPP-46 on SPP1-induced upregulation of pro-inflammatory gene expression in macrophages, assessed via qPCR. (**C**) Semi-quantitative analysis of the suppressive effect of TEPP-46 on SPP1-induced pro-inflammatory protein expression in macrophages.

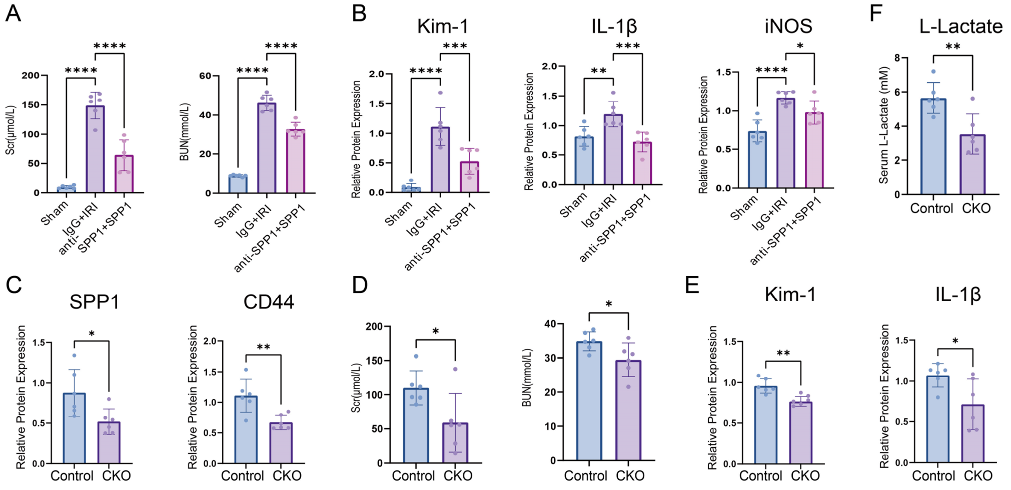

Fig. S6. Targeting or genetically ablating tubular SPP1 attenuates renal IRI (A) Protective effects of SPP1 neutralizing antibody treatment on renal function indices (Scr and BU) in IRI mice. (**B**) Semi-quantitative analysis of renal injury and inflammatory factor protein expression in IRI mice following SPP1 neutralizing antibody intervention. (**C**) Semi-quantitative analysis of knockout efficiency and renal CD44 expression in renal tubular epithelial cell–specific SPP1 conditional knockout mice. (**D**) Protective effects of SPP1 conditional knockout on renal function indices (Scr and BUN) in IRI mice. (**E**) Semi-quantitative analysis of renal injury and inflammation-related protein expression in IRI mice following SPP1 conditional knockout. (**F**) Quantitative analysis of serum lactate levels in IRI mice following SPP1 conditional knockout.
